# Oral Glucosamine in the Treatment of Temporomandibular Joint Osteoarthritis: A Systematic Review

**DOI:** 10.3390/ijms24054925

**Published:** 2023-03-03

**Authors:** Marcin Derwich, Bartłomiej Górski, Elie Amm, Elżbieta Pawłowska

**Affiliations:** 1 Department of Pediatric Dentistry, Medical University of Lodz, 90-419 Łódź, Poland; 2 Department of Periodontology and Oral Mucosa Diseases, Medical University of Warsaw, 02-097 Warsaw, Poland; 3 Department of Orthodontics and Dentofacial Orthopedics, Boston University, Boston, MA 02118, USA

**Keywords:** glucosamine, temporomandibular disorders, temporomandibular joint osteoarthritis, temporomandibular joints

## Abstract

Temporomandibular disorders (TMDs) occur frequently within the general population and are the most common non-dental cause of orofacial pain. Temporomandibular joint osteoarthritis (TMJ OA) is a degenerative joint disease (DJD). There have been several different methods of treatment of TMJ OA listed, including pharmacotherapy among others. Due to its anti-aging, antioxidative, bacteriostatic, anti-inflammatory, immuno-stimulating, pro-anabolic and anti-catabolic properties, oral glucosamine seems to be a potentially very effective agent in the treatment of TMJ OA. The aim of this review was to critically assess the efficacy of oral glucosamine in the treatment of TMJ OA on the basis of the literature. PubMed and Scopus databases were analyzed with the keywords: (temporomandibular joints) AND ((disorders) OR (osteoarthritis)) AND (treatment) AND (glucosamine). After the screening of 50 results, eight studies have been included in this review. Oral glucosamine is one of the symptomatic slow-acting drugs for osteoarthritis. There is not enough scientific evidence to unambiguously confirm the clinical effectiveness of glucosamine supplements in the treatment of TMJ OA on the basis of the literature. The most important aspect affecting the clinical efficacy of oral glucosamine in the treatment of TMJ OA was the total administration time. Administration of oral glucosamine for a longer period of time, i.e., 3 months, led to a significant reduction in TMJ pain and a significant increase in maximum mouth opening. It also resulted in long-term anti-inflammatory effects within the TMJs. Further long-term, randomized, double-blind studies, with a unified methodology, ought to be performed to draw the general recommendations for the use of oral glucosamine in the treatment of TMJ OA.

## 1. Introduction

The term temporomandibular disorders (TMDs) refers to different musculoskeletal and neuromuscular disorders that affect the temporomandibular joints (TMJs), masticatory muscles, and surrounding tissues [1,2]. According to the Diagnostic Criteria for Temporomandibular Disorders (DC/TMD), there are three major groups of TMDs: pain-related TMDs (myalgia, arthralgia, headache attributed to TMD), intra-articular TMDs (disc displacement with reduction, disc displacement with reduction with intermittent locking, disc displacement without reduction with limited opening, disc displacement without reduction without limited opening), and degenerative joint disease (DJD), including temporomandibular joint osteoarthritis (TMJ OA), and subluxation [2]. The TMDs are the most common nondental cause of the orofacial pain [3].

The etiology of TMDs is multifactorial. Within the literature, several different factors have been listed, that may lead to the development of TMDs, including trauma, psychosocial factors, systemic factors (comorbid diseases, i.e., rheumatologic, endocrine, neurologic, myopathies, fibromyalgia, insomnia, irritable bowel syndrome), local factors (nonspecific orofacial symptoms, including stiffness, soreness, cramping), and genetic factors [4]. 

TMDs occur approximately in 31% of adults and in 11% of adolescents [5]. The most common type of TMDs is disc displacement with reduction [5,6]. Although the prevalence of TMDs in the general population is relatively high, only up to 5% of those patients require treatment [7]. 

Patients who suffer from TMDs most often seek treatment because of the pain in the area of the TMJ and/or due to the limited mouth opening. Therefore, the aims of the treatment are to reduce the joint pain, increase the maximum mouth opening, prevent further joint damage, and improve a patient’s overall quality of life [8].

There are several different methods of treatment of TMDs. Those methods may be allocated into one of the three categories, including conservative methods of treatment, minimally invasive surgical procedures, and invasive surgical procedures [9,10,11]. Conservative methods of treatment are the most common ones and encompass counselling, physiotherapy, occlusal splint therapy, and pharmacotherapy [4,9]. Conservative methods of treatment have been found to be effective in pain relief in patients diagnosed with intracapsular TMD [12]. Occlusal splint therapy and physiotherapy, combined together, are currently most often recommended for patients diagnosed with TMD [12,13]. Pharmacotherapy is considered to be the complementary therapy in the treatment of TMDs, especially for pain reduction [9]. The most commonly used drugs in pain alleviation in the area of TMJs are nonsteroidal anti-inflammatory drugs (NSAIDs) [14]. However, there are also some other substances, which may be very beneficial for TMJs. One of those is glucosamine, which presents anti-inflammatory and antioxidative properties, among others [15].

Glucosamine is an amino monosaccharide that is naturally biosynthesized in the human body [16]. Glucosamine is a fundamental component of mucopolysaccharides, glycosaminoglycans. Glycosaminoglycans are attached to the protein core and form proteoglycans, which are part of the extracellular matrix of the articular cartilage [17]. Oral glucosamine is one of the symptomatic slow-acting drugs for osteoarthritis (SYSADOAs) [18]. The European Society for Clinical and Economic Aspects of Osteoporosis, Osteoarthritis and Musculoskeletal Diseases (ESCEO) working group strongly recommended the use of crystalline glucosamine sulfate as a step 1 long-term background treatment for the treatment of knee osteoarthritis [19]. Moreover, they discouraged to use other forms of glucosamine [19]. Within the literature, the use of glucosamines has also been recommended for the treatment of hip osteoarthritis [20,21], as well as hand osteoarthritis [21]. There is also limited evidence that glucosamine sulfate appeared to be potentially beneficial in the treatment of ankle osteoarthritis [22]. Although the abovementioned recommendations seem obvious, they are not as clear when related to the treatment of TMDs, including temporomandibular joint osteoarthritis (TMJ OA).

Therefore, the aim of this systematic review was to critically assess the efficacy of oral glucosamine in the treatment of TMJ OA on the basis of the literature.

## 2. Materials and Methods

### 2.1. Search Strategy

This systematic review was performed with reference to the Preferred Reporting Items for Systematic Reviews and Meta-Analyses (PRISMA) guidelines. PubMed and SCOPUS databases were searched for the randomized clinical trials and randomized clinical studies assessing the efficacy of glucosamine in the treatment of temporomandibular joint osteoarthritis. The databases were searched for manuscripts published in English until 31 December 2022.

The PubMed database was analyzed with the keywords: (“temporomandibular joint” (MeSH Terms) OR (“temporomandibular” (All Fields) AND “joint” (All Fields)) OR “temporomandibular joint” (All Fields) OR (“temporomandibular” (All Fields) AND “joints” (All Fields)) OR “temporomandibular joints” (All Fields)) AND (“disease” (MeSH Terms) OR “disease” (All Fields) OR “disorder” (All Fields) OR “disorders” (All Fields) OR “disorder s” (All Fields) OR “disordes” (All Fields) OR (“osteoarthritis” (MeSH Terms) OR “osteoarthritis” (All Fields) OR “osteoarthritides” (All Fields))) AND (“therapeutics” (MeSH Terms) OR “therapeutics” (All Fields) OR “treatments” (All Fields) OR “therapy” (MeSH Subheading) OR “therapy” (All Fields) OR “treatment” (All Fields) OR “treatment s” (All Fields)) AND (“glucosamin” (All Fields) OR “glucosamine” (MeSH Terms) OR “glucosamine” (All Fields) OR “glucosamines” (All Fields)). 

The SCOPUS database was analyzed with the keywords: ((“temporomandibular joints”) AND ((“disorders”) OR (“osteoarthritis”)) AND (“treatment”) AND (“glucosamine”)). 

### 2.2. Clinical Question

What is the efficacy of orally administered glucosamine in the treatment of the TMJ OA on the basis of the literature?

### 2.3. Selection Criteria

Having removed the duplicates, two of the reviewers (M.D. and B.G.) independently analyzed the manuscripts for eligibility. In the case of disagreement, the abovementioned authors consulted the third reviewer (E.P.). 

We used the PICO approach to properly develop literature search strategies for this review: 

*Population*: 

Adult patients (aged 18 years or more) who were diagnosed with TMJ OA. The diagnosis of the TMJ OA was made on the basis of the DC/TMD diagnostic criteria.

*Intervention*: 

Treatment of TMJ OA with glucosamine administered orally.

*Comparison*: 

Different pharmacological treatments: occlusal splints, placebo, no treatment.

*Outcome*: 

Decreased pain in the TMJ area on the basis of the visual analogue scale (VAS), and increased maximum mouth opening, measured in millimeters (mm). 

We included only randomized clinical trials and randomized clinical studies on adult humans who were diagnosed with TMJ OA. We excluded: (1) animal studies, (2) studies on children, (3) retrospective cohort studies, (4) studies without a control group, (5) articles published in a language other than English, (6) comments, (7) case reports, (8) reviews and meta-analyses.

### 2.4. Cohen’s Kappa Coefficient

The Cohen’s kappa coefficient between the reviewers was 1.00.

### 2.5. Risk of Bias Assessment

Risk of bias of the selected manuscripts was performed following the Cochrane handbook for systematic reviews of interventions version 6.3 (updated February 2022) [23]. Each manuscript was evaluated by two of the reviewers (M.D. and B.G.) independently and in the case of disagreement, the results were consulted by the third reviewer (E.P.).

## 3. Results

Having analyzed the PubMed and Scopus databases, we found 50 references by electronic search. There were 15 duplicates, which were removed. Therefore, 35 records remained. Twenty-seven references did not meet the eligibility criteria and were excluded. Within the excluded records there were: two articles published in a language other than English, 15 reviews, one comment, two animal studies, two studies without a control group, one case report, one retrospective cohort study, and three studies excluded because of other reasons (unclear methodology regarding the use of glucosamine). Finally, there were eight studies included in this systematic review.

**Figure 1:**
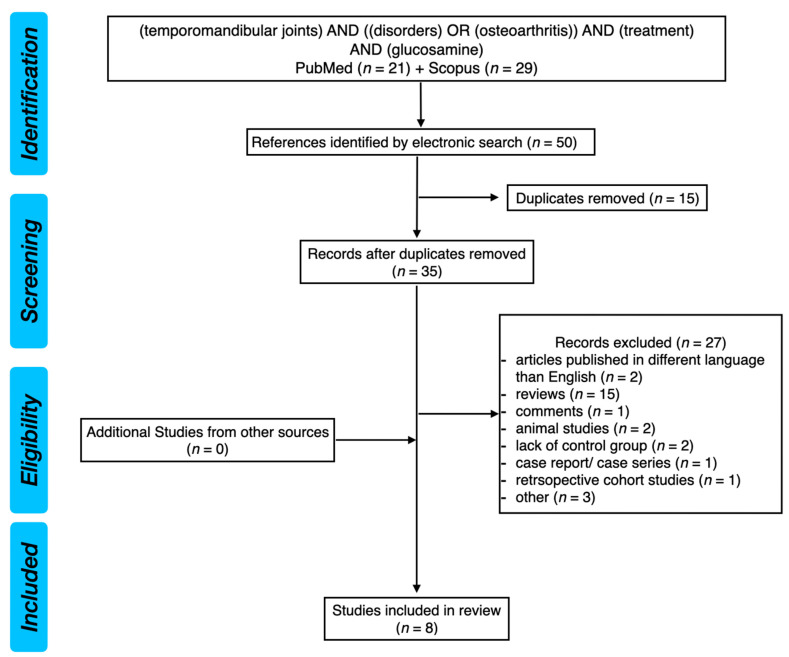
PRISMA flow diagram for the review of the literature.

Figure 1 presents the PRISMA flow diagram for the review of the literature.

The selected studies were published between the years 2001 and 2021. Table 1 presents the effectiveness of the orally administered glucosamine used in the treatment of TMJ OA, on the basis of the studies included in the systematic review [24,25,26,27,28,29,30,31].

Considering the risk of bias, four studies were judged as low risk [24,28,29,30], one as unclear risk [26], and three as high risk [25,27,31]. Table 2 summarizes the quality of the included RCTs.

Glucosamine sulfate administered orally, as the only one method of treatment in patients with TMD, appeared to be superior to ibuprofen in terms of pain reduction in the area of TMJs [24,25], as well as in terms of increasing maximum mouth opening [25]. Although glucosamine sulfate combined with chondroitin sulfate and tramadol led to a significant pain reduction in the area of TMJs, only glucosamine sulfate combined with chondroitin sulfate was successful in increasing maximum mouth opening [26]. When glucosamine was compared only to placebo, it appeared that after 3 months, not only did the patients present decreased pain in the area of TMJs, but also decreased sounds within TMJs, and needed a decreased number of over-the-counter medications [27]. However, in short-term (6 weeks) observation oral glucosamine was not superior to placebo [28]. Patients who were supplemented glucosamine additionally, through intra-articular injections of hyaluronic acid, did not benefit in the short-term, but presented significantly decreased pain levels and significantly increased maximum mouth opening in the long-term (1 year) observations [29,30]. Glucosamine administered additionally to single-session arthrocentesis and the intra-articular injection of HA did not lead to better clinical results than those which were obtained by the patients who had not received oral glucosamine [31]. This confirms that arthrocentesis of TMJs is a very effective, minimally invasive surgical procedure, as previously reported in the literature [11].

## 4. Discussion

The presented systematic review analyzed the efficacy of glucosamine in the treatment of the TMJ OA.

Glucosamine (N-Acetyl-D -glucosamine, C_6_H_13_NO_5_) is a water-soluble monosaccharide composed of a glucose molecule attached to an amino group, which is produced from chitin or chitosan by hydrolysis [32]. It is a derivative of cellular glucose endogenous metabolism in the body, in which the hydroxyl group in the 2-position has been replaced with an amino group. With respect to the nomenclature created and developed by the International Union of Pure and Applied Chemistry (IUPAC), the full chemical name of glucosamine is (3R,4R,5S)-3-amino-6-(hydroxymethyl)oxane-2,4,5-triol [33].

Glucosamine is a natural constituent of glycosaminoglycans and proteoglycans of the cartilage matrix covering the epiphyseal parts of bones. It is also a vital component of hyaluronic acid, which forms synovial fluid within the joint. The main source of exogenous glucosamine is the exoskeleton of crustaceans. In humans, glucosamine occurs in several dosage forms: glucosamine sulfate (GS), glucosamine hydrochloride (GH), which does not have a sulfate group and crystalline glucosamine sulfate [34]. GS needs compound stabilizers in the form of salts, usually potassium chloride (KCl) or sodium chloride (NaCl), hence it has a 74% purity. GH, on the other hand, has a 99% purity. Consequently, a dosage of 1500 mg of GH equals a dosage of 2608 mg of GS [35].

Table 3 presents the comparison of the selected properties of glucosamine sulfate and glucosamine hydrochloride on the basis of the literature.

Table 4 presents exemplary orally administered glucosamine sulfate and glucosamine hydrochloride available in Poland, on the basis of Pharmindex [43].

Glucosamine can be extracted and chemically stabilized for oral administration. Generic, over-the-counter formulations, and food supplements mostly contain glucosamine hydrochloride salt, while patented crystalline glucosamine sulfate (pCGS) formulation is available only on prescription [44]. After oral administration, glucosamine is easily absorbed from the gastrointestinal tract. Both the GS and GH dissociate in the acidic milieu of the stomach, resulting in the release of glucosamine itself [45]. It undergoes metabolism in the liver and the first pass effect. Subsequently, it is eliminated in the feces and urine. Glucosamine displays protein binding as high as 90%, thus it reaches the peak level following oral intake after 8 h [46]. Three hours after oral intake of a dose of 1500 mg of GS, the maximal plasma concentration was 10 μM and it reached a steady state [38]. The elimination half-life was estimated to be 15 h. In another study, GS reached constant and higher levels (up to 25% higher) in the synovial fluid compared to plasma [47]. A single oral administration of 1500 mg of GH led to a maximal plasma concentration of 492 ± 161 ng/mL reached in 2.31 ± 1.19 h [39]. Chondroitin sulfate inhibited GH absorption and decreased its biodisponibility.

**Figure 2:**
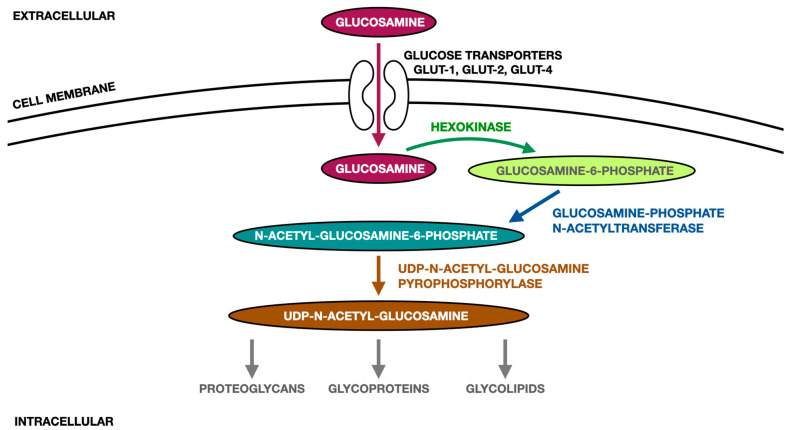
Metabolic pathway of orally administered glucosamine on the basis of the literature [46].

It is estimated that nearly 90% of orally taken glucosamine is absorbed [48]. Glucosamine is transported into the human cells primarily via insulin-dependent glucose transporters (GLUT-2 and GLUT-4). It is also possible to transport glucosamine via glucose transporter GLUT-1. Within the cell, glucosamine becomes phosphorylated to glucosamine-6-phosphate, and subsequently acetylated to N-acetyl-glucosamine-6-phosphate. Finally, the previously mentioned compound is transformed into uridine-5-diphosphate-N-acetyl-glucosamine, which takes part in the biosynthesis of amino sugars, which are used as units for glycosaminoglycans, proteoglycans and glycoproteins [46,49]. Figure 2 presents the metabolic pathway of orally administered glucosamine on the basis of the literature [46].

Several research studies have provided data on anti-aging, antioxidative, bacteriostatic, anti-inflammatory, immuno-stimulating, pro-anabolic and anti-catabolic mechanisms of action of glucosamine [15]. Moreover, antifibrotic, neuroprotective, cardioprotective, anticancer and antineoplastic properties of glucosamine were also reported [50]. The antioxidant activity of glucosamine was also reported. In an in vitro study, GH showed multiple antioxidant activities, such as considerable reducing power, superoxide/hydroxyl radical scavenging ability, and ferrous ion chelating potency [41]. 

Thanks to its antioxidative, anti-inflammatory, and immuno-stimulating properties especially, glucosamine has been used in the treatment of TMJ OA.

Thie et al. [24] compared the clinical effects of the use of glucosamine sulfate (500 mg) and ibuprofen (400 mg) taken for 3 months (90 days) in patients diagnosed with degenerative joint disease (DJD). DJD was diagnosed on the basis of computed tomography (CT) images. Patients were randomly allocated into one of the groups. Before the onset of the study, all of the patients underwent a one-week washout period. Patients were allowed to take acetaminophen 500 mg in case of pain (maximum 4000 mg/day). Both groups presented a statistically significant decrease in pain levels in the area of TMJs, as well as a statistically significant increase in maximum mouth opening (both pain-free and voluntary) after the end of the treatment. The authors noticed that six patients from the glucosamine group and seven patients from the ibuprofen group did not achieve 20% improvement in TMJ functional pain. Having compared the effects of glucosamine and ibuprofen in patients who obtained at least 20% reduction in TMJ functional pain, it appeared that patients who received glucosamine presented a statistically significant improvement of functional pain evaluation and overall pain interference compared to patients who received ibuprofen. Finally, patients from the glucosamine group did not need to take as many acetaminophen tablets between Day 90 and 120 as the patients who received ibuprofen. Thie et al. [24] concluded that oral glucosamine was at least as effective as the traditional pharmaceuticals used in the treatment of TMD. Although, the results presented by Thie et al. [24] are very promising, it may be speculated that acetaminophen intake in both groups could have significantly affected the final results. Moreover, ibuprofen in a dosage of 400 mg, taken three times per day for 90 days, significantly increases the risk of gastrointestinal and cardiovascular complications. Therefore, it is not recommended to take NSAIDs for longer than 14 days [14].

Haghighat et al. [25] also compared the efficacy of glucosamine sulfate (1500 mg daily for 90 days) versus ibuprofen (400 mg twice a day for 90 days) in patients with TMJ pain, crepitation, or limited mouth opening. The authors found that patients taking oral glucosamine presented a significantly lower TMJ pain intensity during all follow-up appointments (30, 60 and 90 days after the onset of the therapy) and significantly increased mandibular opening following the end of the second month of treatment (60 and 90 days after the onset of the therapy) compared to patients being administered ibuprofen. The authors recommended using oral glucosamine as a more effective and safer drug compared to ibuprofen for the treatment of the examined TMDs.

Damlar et al. [26] performed a randomized clinical trial and enrolled patients diagnosed with TMJ internal derangement (Wilkes II or Wilkes III). All the patients had their joints rinsed with 2 mL of saline solution (repeated 10 times) twice: before and after the end of the study. Patients were randomly allocated into either the study group (1500 mg glucosamine sulfate and 1200 mg chondroitin sulfate per day) or the control group (50 mg tramadol 2× per day for pain control). Both groups presented significantly reduced pain levels after the end of the treatment, whereas significantly increased maximum mouth opening was observed only in the study group. Having compared the changes that occurred within both of the examined groups, the study group presented significantly decreased concentration of IL-1β and IL-6.

Nguyen et al. [27] randomly allocated the participants into one of the subgroups: the active medication group, where patients took three tablets twice a day for three months (per tablet: glucosamine hydrochloride 250 mg and chondroitin sulfate 200 mg); and the inactive medication group, where patients received three tablets of placebo twice a day for three months. Some of the patients received an injection of 1.8 mL of lidocaine 1% into the lateral pterygoid region or the temporal tendon during the initial appointment. Some of them were also prescribed jaw stretch exercises. Those additional methods of treatment were not repeated until the end of the study. Nguyen et al. [27] noticed that patients who received glucosamine hydrochloride with chondroitin sulfate presented decreased tenderness in the area of TMJs, decreased sounds within TMJs, and needed a decreased number of over-the-counter medications. Interestingly, patients who received the placebo, reported significantly decreased pain within the TMJs after the end of the treatment.

Cahlin et al. [28] conducted a short-term study comparing the effectiveness of glucosamine sulfate (400 mg per capsule, three capsules per day for 6 weeks) versus placebo in the treatment of TMJ OA. Only patients who had been administered oral glucosamine presented a significant pain reduction (based on the visual analog scale), as well as significant increase in opening capacity without and with pain. However, comparing the results obtained after 6 weeks, there were no statistically significant differences between the groups. Therefore, the authors concluded that the effectiveness of oral glucosamine sulfate in TMJ pain reduction, as well as in the reduction of osteoarthritis symptoms was not better than the placebo. However, it must be emphasized, that this study was a short-term one. Other studies [29,30] that assessed both the short-term and long-term efficacy of oral glucosamine supplementation also found that its short-term effectiveness was similar to the placebo.

Cen et al. [29] performed their study to find out whether oral glucosamine supplementation, additionally to intra-articular injections of hyaluronic acid (HA), is beneficial for patients diagnosed with TMJ osteoarthritis (TMJ OA). The authors randomly allocated 136 patients into one of the two subgroups. Patients from the intervention group underwent intra-articular injections (1 mL sodium HA injected into the superior and inferior joint spaces) performed once a week for 4 following weeks. Moreover, they were prescribed two tablets of GS hydrochloride (240 mg) three times a day for 3 months. Patients from the control group received the same protocol of treatment, but instead of GS hydrochloride (240 mg) they were given placebo tablets (240 mg). Before the intra-articular injection of HA, all of the superior joint spaces were rinsed three times with 2 mL of saline solution. In the short-term (1 month) observation, both groups presented a statistically significant increase of maximum mouth opening and a statistically significant pain reduction. There were no statistically significant differences between the examined groups regarding the maximum mouth opening and pain levels. The concentrations of interleukin 1β (IL-1β) and interleukin 6 (IL-6) in the TMJ synovial fluid significantly decreased in both groups (the concentration of IL-6 decreased significantly more in the intervention group). Although, the concentration of transforming growth factor beta (TGF-β) significantly increased in the intervention group, the difference between the groups was statistically insignificant. The authors also noticed that patients younger than 45 years presented significantly larger maximum mouth opening, a significantly lower pain score, and a significantly lower concentration of IL-1β after one month of the treatment. Interestingly, in the long-term observation (1 year), patients who received oral glucosamine presented significantly increased maximum mouth opening, significantly decreased pain scores, significantly increased the concentration of TGF-β and significantly decreased the concentration of IL-1β and IL-6 compared to the control group. However, it must be noted that the concentration of IL-6 increased in both groups up to values similar to those from the onset of the treatment. There were no differences in the measurements regarding the age of the patients. Long-term administration of oral glucosamine appeared to be beneficial for the patients.

A similar double-blind, randomized, controlled trial was conducted by Yang et al. [30]. They allocated patients with TMJ OA randomly into one of the following subgroups: TMJ hyaluronate sodium intra-articular injection with oral glucosamine supplementation (glucosamine hydrochloride 240 mg every 8 h for 3 months) and TMJ hyaluronate sodium intra-articular injection with placebo pills. Yang et al. [30] found that additional oral glucosamine supplementation in the short term had no extra effect on TMJ OA compared to the TMJ hyaluronate sodium intra-articular injection alone; whereas, in the long-term observations, complementary administration of oral glucosamine led to a significant pain reduction and significant increase in maximum mouth opening. Therefore, their results confirmed observations by Cen et al. [29]. However, both of the abovementioned studies [29,30] were conducted at the same Sichuan University (Chengdu, China).

Kılıç [31] performed a study in which he analyzed different methods of treatments of patients with TMJ OA. The patients were randomly allocated into one of the groups: control group (single-session arthrocentesis + intra-articular injection of HA) or study group (single-session arthrocentesis + intra-articular injection of HA + 3 months of supplementation of: 750 mg glucosamine hydrochloride, 600 mg chondroitin sulfate, and 350 mg methylsulfonylmethane, 2 × 1 dosage daily). The results obtained in both groups were similar. Pain complaints and joint sounds significantly decreased, whereas masticatory efficiency and lateral mandibular mobility significantly increased in both groups after the end of the treatment. Maximum mouth opening (with or without pain) and mandibular protrusive motion did not change significantly in any of the examined groups. Both methods of treatment presented similar effectiveness.

A few limitations to this systematic review can be listed. Firstly, there were only four out of eight manuscripts which were assessed as low risk of bias. Secondly, the included studies presented different methodologies. Some authors used glucosamine as the only method of treatment, whereas the others combined intra-articular injections with oral glucosamine supplementation. Moreover, although all of the patients presented degenerative joint disease, some of the authors also included patients who had also been diagnosed with disc dislocation, disc displacement or cellulitis into the study. Thirdly, the authors used different compounds of glucosamine: some of them used glucosamine sulfate, whereas the others used glucosamine hydrochloride. Although both compounds are very similar, their clinical efficacy in the treatment of TMJ OA ought to be compared in the future studies. 

## 5. Conclusions

There is not enough scientific evidence to unambiguously confirm the clinical effectiveness of glucosamine supplements in the treatment of TMJ OA on the basis of the literature. The most important aspect affecting the clinical efficacy of oral glucosamine in the treatment of TMJ OA was the total administration time. Administration of oral glucosamine for a longer period of time, i.e., 3 months, led to a significant reduction of TMJ pain and a significant increase in maximum mouth opening. It also resulted in long-term anti-inflammatory effects within the TMJs. 

Further long-term, randomized, double-blind studies, with a unified methodology, ought to be performed to draw the general recommendations for the use of oral glucosamine in the treatment of TMJ OA.

## Figures and Tables

**Table 1:** Effectiveness of the orally administered glucosamine used in the treatment of TMJ OA, on the basis of the studies included in the systematic review [24,25,26,27,28,29,30,31].

Reference	Study Design	Participants and Intervention	Endpoint and Results
Thie et al. (2001) [24]	Randomized, double-blind study	45 patients (40 women, 5 men) qualified for the study, 39 patients completed the study *Diagnosis:* degenerative joint disease in at least one of the TMJs *Imaging of TMJs:* CT *Study groups:* - glucosamine sulfate 500 mg tablets taken q8h (21 patients, mean age: 36.62 ± 10.3 years) - ibuprofen 400 mg tabelts taken q8h (18 patients, mean age: 38.73 ± 13.3 years) - all of the patients were also allowed to take acetaminophen 500 mg for pain breakthrough	*Endpoint*: 3 months (90 days) Both the ibuprofen and glucosamine led to a significant reduction in pain levels in patients with DJD. Patients who received glucosamine presented statistically significant improvement of functional pain evaluation and an overall pain interference compared to patients who received ibuprofen.
Haghighat et al. (2013) [25]	Randomized clinical trial	60 patients (46 women, 14 men) *Diagnosis:* painful TMJ, TMJ crepitation, limitation of mouth opening *Imaging of TMJs:* none *Study groups:* - glucosamine sulfate 1500 mg daily for 90 days (30 patients, mean age: 26.60 ± 10.32 years) - ibuprofen 400 mg 2×/day for 90 days (30 patients, mean age: 27.12 ± 10.83 years)	*Endpoint*: 90 days Patients taking oral glucosamine presented significantly lower TMJ pain intensity during all follow-up appointments and significantly increased mandibular opening following the end of the second month of treatment. Oral glucosamine was recommended as a more effective and safer drug compared to ibuprofen for the treatment of TMJ OA.
Damlar et al. (2015) [26]	Randomized clinical trial	34 patients (34 women, mean age: 28.6 ± 6.89 years) *Diagnosis:* TMJ internal derangement (Wilkes II or III) *Imaging of TMJs:* MRI *Study groups:* - study group: 1500 mg glucosamine sulfate and 1200 mg chondroitin sulfate per day (16 patients) - control group: 50 mg tramadol HCl 2× daily peroral for pain relief (15 patients) - all of the superior and inferior joint spaces were rinsed with 2 mL of saline solution (procedure repeated 10 times)	*Endpoint*: 8 weeks Both groups presented significantly reduced pain levels after the end of the treatment, whereas significantly increased maximum mouth opening was observed only in the study group. Having compared the changes that occurred within both of the examined groups, the study group presented a significantly decreased concentration of IL-1β and IL-6.
Nguyen et al. (2001) [27]	Randomized, double-blind study	34 patients (30 women, 4 men) *Diagnosis:* cellulitis, disc displacement, disc dislocation, painful osteoarthritis of the TMJ *Imaging of TMJs:* no information *Study groups:* - the active medication group (CS-GH): 3 tablets twice a day for three months, containing glucosamine hydrochloride 250 mg and chondroitin sulfate 200 mg (14 patients, mean age 43 ± 14 years) - the inactive medication group (placebo): 3 tablets twice a day for three months, containing placebo (20 patients, mean age 46 ± 15 years)	*Endpoint*: 3 months Patients CS-GH presented decreased tenderness in the area of TMJs, decreased sounds within TMJs, and needed a decreased number of over-the-counter medications. Patients who received placebo, reported a significantly decreased pain within the TMJs after the end of the treatment.
Cahlin et al. (2011) [28]	Randomized, double-blind study	59 patients (51 women, 8 men) *Diagnosis:* presence of osteoarthritis in at least one of the TMJs *Imaging of TMJs:* lack of precise information *Study groups:* - the glucosamine sulfate group (glucosmaine sulfate 400 mg): 3 capsules a day for six weeks (30 patients, mean age female: 61 ± 16 years, mean age male: 61 ± 9 years) - the placebo group (placebo 400 mg): 3 capsules a day for six weeks (29 patients, mean age female: 59 ± 8 years, mean age male: 49 ± 11 years) - all of the patients were given 15 tablets of paracetamol (at 1000 mg) as a rescue medication (patients could ask for more tablets if needed)	*Endpoint*: 6 weeks The effectiveness of oral glucosamine sulfate in TMJ pain reduction, as well as in reduction of TMJ osteoarthritis symptoms, was not better than the placebo.
Cen et al. (2018) [29]	Randomized, double-blind study	136 patients (118 women, 18 men) *Diagnosis:* TMJ osteoarthritis *Imaging of TMJs:* CBCT *Study groups:* - the intervention group (GS + HA): 1 mL sodium HA injection into the superior and inferior spaces of TMJ, repeated in total 4 times (once a week for 4 following weeks) + 2 tablets of glucosamine hydrochloride 240 mg taken 3 times a day for 3 months (67 patients, mean age 40.1 ± 15.8 years) - the control group (placebo + HA): 1 mL sodium HA injection into the superior and inferior spaces of TMJ, repeated in total 4 times (once a week for 4 following weeks) + 2 tablets of placebo 240 mg taken 3 times a day for 3 months (69 patients, mean age 36.2 ± 15.8 years) - all of the superior joint spaces were rinsed with 2 mL of saline solution (3 times) prior to HA intra-articular injection	*Endpoint*: 1 year In the short-term (1 month) observation, both groups presented a statistically significant increase in maximum mouth opening, statistically significant pain reduction, and significantly decreased concetration of IL-1β and IL-6 (no differences between the groups aparat from the concentartion of IL-6, which was significantly lower in intervention group). After 1 year, patients who received oral glucosamine presented significantly increased maximum mouth opening, significantly decreased pain scores, significantly increased concentration of TGF-β and significantly decreased concentration of IL-1β and IL-6 compared to the control group.
Yang et al. (2018) [30]	Randomized, double-blind study	144 patients (120 women, 24 men) *Diagnosis:* TMJ osteoarthritis *Imaging of TMJs:* CBCT *Study groups:* - group A: 4 intraraticular injections of 2 mL sodium hyaluronate 1×/week + 2 tablets of glucosamine hydrochloride 240 mg 3×/day for 3 months (72 patients, mean age: 40.1 ± 15.8 years) - group B: 4 intra-articular injection of 2 mL sodium hyaluronate 1×/week + placebo pills for 3 months (72 patients, mean age: 36.2 ± 15.8 years) - all of the superior and inferior joint spaces were rinsed prior to HA intra-articular injection - all of the patients received Diclofenac (50 mg) as a rescue medication	*Endpoint*: 1 year Oral glucosamine supplementation, in short term, had no extra effect on TMJ OA; whereas, in the long-term observations, complementary administration of oral glucosamine led to significant TMJ pain reduction and significant increase in maximum mouth opening.
Kılıç (2021) [31]	Randomized clinical trial	26 patients (23 women, 3 men) *Diagnosis:* TMJ osteoarthritis *Imaging of TMJs:* CBCT *Study groups:* - group 1 (control): single-session arthrocentesis + intra-articular injection of HA (14 patients, mean age: 28.71 ± 10.94 years) - group 2 (study): single-session arthrocentesis + intra-articular injection of HA + 3 months of supplementation of 750 mg glucosamine hydrochloride, 600 mg chondroitin sulfate, and 350 mg methylsulfonylmethane (2 × 1 dosage daily) (12 patients, mean age: 27.92 ± 11.20 years)	*Endpoint*: 1 year The results obtained in both groups were similar. Both methods of treatment presented a similar effectiveness.

CBCT—cone beam computed tomography, CS-GH—chondroitin sulfate-glucosamine hydrochloride, CT—computed tomography, DJD—degenerative joint disease, G/D—glucosamine/diclofenac, GS—glucosamine, HA—hyaluronic acid, IL-1β—interleukin 1 beta, IL-6—interleukin 6, MRI—magnetic resonance imaging, q8h—every 8 h, TMJs—temporomandibular joints, TGFβ—transforming growth factor beta.

**Table 2:** Risk-of-bias assessment of selected studies following the Cochrane Handbook for Systematic Review of Interventions.

	Random Sequence Generation (Selection Bias)	Allocation Concealment (Selection Bias)	Blinding of Participants (Performance Bias)	Blinding of Outcome Assessment (Detection Bias)	Incomplete Outcome Data (Attrition Bias)	Selective Reporting (Reporting Bias)	Other Bias
Thie et al. (2001) [24]	+	+	+	+	+	+	?
Haghighat et al.(2013) [25]	?	?	–	–	+	+	+
Damlar et al. (2015) [26]	?	+	+	+	+	+	+
Nguyen et al. (2001) [27]	+	+	+	?	-	+	+
Cahlin et al. (2011) [28]	+	+	+	+	+	+	+
Cen et al. (2018) [29]	+	+	+	+	+	+	+
Yang et al. (2018) [30]	+	+	+	+	+	+	+
Kılıç (2021) [31]	+	?	–	+	+	+	+

“+” = without bias, “–” = with bias, “?” = no information.

**Table 3:** Comparison of the selected properties of glucosamine sulfate and glucosamine hydrochloride on the basis of the literature.

Variables	Glucosamine Sulfate (GS)	Glucosamine Hydrochloride (GH)	References
Molecular structure	C_6_H_15_NO_9_S	C_6_H_14_ClNO_5_	[36,37]
Molecular weight [unit]	277.25	215.63	[36,37]
Stability and purity	Needs compound stabilizers in the form of salts, usually potassium chloride (KCl) or sodium chloride (NaCl). It has a 74% purity.	Does not need compound stabilizers. It has a 99% purity.	[38,39]
Pharmacokinetic parameters when administrated 1500 mg once daily steady-state	C_max_ (mean) 1602 ± 425 ng/mL 8.9 ± 2.4 μmol/L T 1/2 (h) 15	C_max_ (mean) 492 ± 161 ng/mL 2.7 ± 0.9 μmol/L T 1/2 (h) 2.51 ± 1.84	[38,39]
Expression of catabolic and anabolic genes	Stronger inhibition	Weaker inhibition	[40]
Antioxidant potential	Pronounced reducing power, superoxide/hydroxyl radical scavenging ability, quite weak ferrous ion chelating effect.	Considerable reducing power, superoxide/hydroxyl radical scavenging ability, limited ferrous ion chelating potency.	[41,42]

C_max_—maximum/peak concentration, T1/2—half-life.

**Table 4:** Exemplary orally administered glucosamine sulfate and glucosamine hydrochloride available in Poland, on the basis of Pharmindex [43].

Exemplary Medicinal Product (ex. Brand Name)	Oral Dosage (Only for Adults)	Maximum Daily Dose	Additional Information
Glucosamine sulfate (Arthryl)	1500 mg once a day	1500 mg	Glucosamine is not used in children and adolescents under 18 years of age and should not be taken during pregnancy and breast-feeding. Glucosamine can be combined with NSAIDs and analgesics in case of the exacerbation of the symptoms.
Glucosamine hydrochloride (Flexove)	625 mg × 2 once a day	1250 mg	Glucosamine is not used in children and adolescents under 18 years of age and should not be taken during pregnancy and breast-feeding. It may increase the effect coumarin anticoagulants (i.e., warfarin).
Chondroitin sulfate sodium and Glucosamine hydrochloride (Chronada)	2 capsules 3 times a day 1 capsule contains: 200 mg Chondrotine sulfate + 250 mg Glucosamine hydrochloride	1200 mg CS + 1500 mg GH	Chronada is not used in children and adolescents under 18 years of age and should not be taken during pregnancy and breast-feeding. It may increase the effect coumarin anticoagulants (i.e., warfarin) or antibiotics (i.e., tetracyclines).

CS—chondroitin sulfate, GH—glucosamine hydrochloride.

## Data Availability

The data underlying this article are available in the article.
